# Aberrant chimeric RNA *GOLM1-MAK10* encoding a secreted fusion protein as a molecular signature for human esophageal squamous cell carcinoma

**DOI:** 10.18632/oncotarget.1465

**Published:** 2013-11-01

**Authors:** Hao Zhang, Wan Lin, Kalpana Kannan, Liming Luo, Jing Li, Pei-Wen Chao, Yan Wang, Yu-Ping Chen, Jiang Gu, Laising Yen

**Affiliations:** ^1^ Department of Integrative Oncology, Affiliated Cancer Hospital, Shantou University Medical College, Shantou, Guangdong, China; ^2^ Cancer Research Center, Shantou University Medical College, Shantou, Guangdong, China; ^3^ Tumor Tissue Bank, Affiliated Cancer Hospital, Shantou University Medical College, Shantou, Guangdong, China; ^4^ Department of Pathology & Immunology, Baylor College of Medicine, Houston, TX, USA; ^5^ Department of Molecular and Cellular Biology, Baylor College of Medicine, Houston, TX, USA; ^6^ Department of Pathology, Shantou University Medical College, Shantou, Guangdong, China; ^7^ Department of Thoracic Surgery, Affiliated Cancer Hospital, Shantou University Medical College, Shantou, Guangdong, China

**Keywords:** Transcriptome, transcription-induced chimeric RNAs, GOLM1-MAK10, secreted fusion protein, cancer biomarker, esophageal cancer

## Abstract

It is increasingly recognized that chimeric RNAs may exert a novel layer of cellular complexity that contributes to oncogenesis and cancer progression, and could be utilized as molecular biomarkers and therapeutic targets. To date yet no fusion chimeric RNAs have been identified in esophageal cancer, the 6th most frequent cause of cancer death in the world. While analyzing the expression of 32 recurrent cancer chimeric RNAs in esophageal squamous cell carcinoma (ESCC) from patients and cancer cell lines, we identified *GOLM1-MAK10*, as a highly cancer-enriched chimeric RNA in ESCC. *In situ* hybridization revealed that the expression of the chimera is largely restricted to cancer cells in patient tumors, and nearly undetectable in non-neoplastic esophageal tissue from normal subjects. The aberrant chimera closely correlated with histologic differentiation and lymph node metastasis. Furthermore, we demonstrate that chimera *GOLM1-MAK10* encodes a secreted fusion protein. Mechanistic studies reveal that *GOLM1-MAK10* is likely derived from transcription read-through/splicing rather than being generated from a fusion gene. Collectively, these findings provide novel insights into the molecular mechanism involved in ESCC and provide a novel potential target for future therapies. The secreted fusion protein translated from *GOLM1-MAK10* could also serve as a unique protein signature detectable by standard non-invasive assays. These observations are critical as there is no clinically useful molecular signature available for detecting this deadly disease or monitoring the treatment response.

## INTRODUCTION

Esophageal squamous cell carcinoma (ESCC) is the third most common malignancy of the digestive tracts [1, 2]. Patients with ESCC usually present with disease that is locally advanced or already metastasized at the time of diagnosis, and are frequently resistant to current chemoradiotherapeutic strategies, resulting in sixth leading cause of cancer death in the world [3]. This is largely due to the limited understanding on molecular mechanisms driving carcinogenesis and progression of this cancer, and the lack of molecular signatures useful for early diagnosis and monitoring therapeutic efficiency.

**Figure 1:**
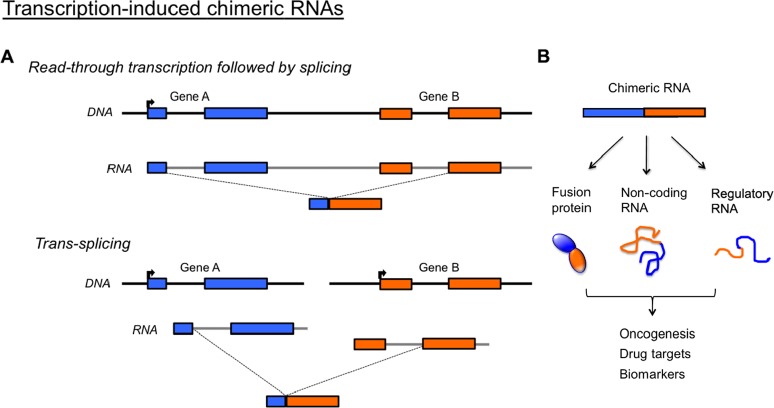
Potential importance of transcription-induced chimeric RNAs (A) Chimeric RNAs unrelated to chromosomal rearrangements are generated through either ‘readthrough/splicing’ or ‘trans-splicing’. Both mechanisms result in fused transcripts that possess sequences from two parental genes. (B) Such chimeric RNA can give rise to novel fusion proteins, non-coding RNA or regulatory RNA that may be responsible for oncogenesis, and serve as drug targets or useful clinical biomarkers. Primer pairs can be specifically designed to amplify chimeric RNA in patients' tissue without amplifying the parental genes.

It is increasingly recognized that chimeric RNAs may exert a novel layer of cellular complexity that contributes to oncogenesis and cancer progression. With the aid of high-throughput RNA sequencing technology and bioinformatics analysis, transcriptome sequencing has uncovered a large number of chimeric RNAs across different human tissues and diseases [4-9]. Studies provide encouraging evidence that signature chimeric RNAs could be identified for the early detection of tumors [6-8]. These chimeric RNAs, which are unrelated to chromosomal rearrangements (fusion genes), are primarily generated by two transcription-induced mechanisms [4, 5] (Figure 1). The first is ‘read-through/splicing’, where a chimeric RNA begins at the upstream gene and ends at a termination point of a downstream gene, with the region in between removed by splicing. The second is ‘trans-splicing’, where two separately transcribed RNAs are spliced together to give rise to a single RNA. Both mechanisms result in fused transcripts that possess sequences from two parental genes.

We recently identified 2,369 chimeric RNA candidates by performing high-throughout sequencing analysis of prostate cancer samples, and confirmed 32 highly cancer-enriched chimeric RNAs [6]. The chimeric RNAs are significant in several ways. First, many are highly enriched in tumor and recurrent among patients [6]. Second, some are released in body fluids such as urine at detectable levels [7, 8], and thus could serve as non-invasive biomarkers for PCR-based assay. Third, chimeric RNAs can lead to the translation of fusion proteins [9], hence potential therapeutic targets (Figure 1). An example is the *BCR-ABL* fusion protein in chronic myeloid leukemia that is now successfully treated by the small molecule drug Gleevec [10]. Despite their importance, chimeric RNAs are largely under-investigated and mostly unknown in esophageal carcinoma.

In this study, we investigated a set of 32 highly recurrent chimeric RNAs recently identified in prostate cancer [6], and characterized their presence in multiple ESCC and non-neoplastic cell lines, in addition to a large cohort of clinical ESCC patient samples. This led to the identification of *GOLM1-MAK10* as highly differentially expressed chimeric RNAs in ESCC. Importantly, the expression of the chimera is markedly elevated in cancer cells within patients' tumor and nearly undetectable in esophageal tissue from subjects without esophageal neoplasia. The aberrant chimera closely correlated with tumor differetiation and lymph node metastasis. Moreover, we showed that the chimera *GOLM1-MAK10* led to the translation of a fusion protein that was secreted into the cell-culture media. Our results thus suggest that the aberrant chimeric RNA *GOLM1-MAK10* is abundant and may represent a novel molecular alteration in ESCC that could have important implications in understanding the mechanism and early detection of ESCC.

## RESULTS

### Identification of *GOLM1-MAK10* in ESCC

**Figure 2:**
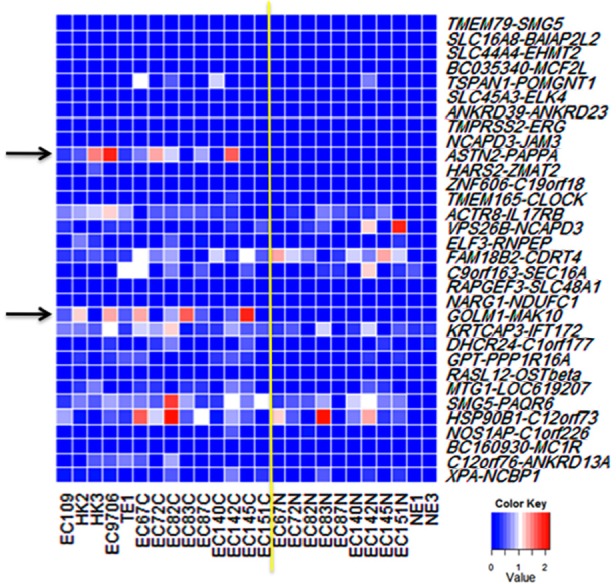
Screening chimeric RNA candidates Initial screening of 32 chimeric RNAs in ESCC cell lines and patients' tissue was performed by RT-PCR using chimera-specific primers. The expression level was graded based on RT-PCR band intensity. The yellow line demarcates cancer from normal. Left side: ESCC cell lines (EC109, HK2, HK3, EC9706, TE1) and cancer tissues. Right side: matched benign tissues and the immortalized normal esophageal epithelial cells (NE1 and NE3). Most of the chimeric RNAs displayed varying expression levels. *ASTN2-PAPPA* and *GOLM1-MAK10*, as marked by arrows, were conspicuously expressed at higher levels in cancer.

**Figure 3:**
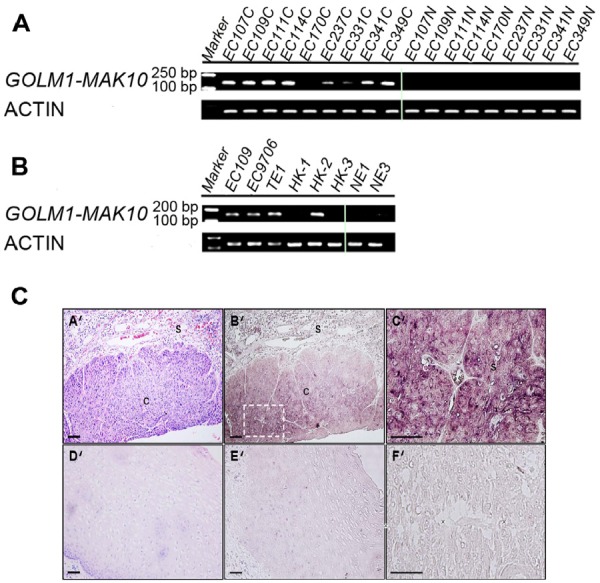
Expression and cellular origin of *GOLM1-MAK10* in ESCC patient tissues and cancer cell lines (A) GOLM1-MAK10 was readily detectable by RT-PCR, and differentially expressed in cancer vs. matched benign tissue. The expression of chimeric RNA in 9 patients are shown. Left side: cancer tissues. Right side: matched benign tissues. (B) GOLM1-MAK10 was expressed in some ESCC cell lines, including EC109, EC9706, TE1, HK1, HK2, and HK3, but minimally expressed in immortalized esophageal epithelial cells NE1 and NE3. Both (A) and (B) suggest that GOLM1-MAK10 is enriched in cancer cells. (C) Cellular origin of *GOLM1-MAK10* RNA in tissue sections of ESCC patient as determined by *in situ* hybridization. (A'), (B'), (C'), and (F'): cancerous esophageal epithelium. (D') and (E'): non-neoplastic esophageal epithelium. Hematoxylin/eosin staining identifies cancer mass with intense purple color in cancerous esophageal epithelium (A'), which is largely absent in non-neoplastic esophageal epithelium (D'). Hybridization with antisense RNA probe reveals the location of chimeric RNAs with dark maroon color in cancerous esophageal epithelium (B'). Note that the areas positive for chimeric RNAs in B' co-localize with cancer mass in AB'. Stromal cells surrounding tumor tissue also showed slight elevated staining but to a lesser extent. In contrast, non-neoplastic esophageal epithelium shows little or no staining with antisense probe (E'). The inset in B' is magnified as C', which reveals the cytoplasmic localization of chimeric RNAs in cancer cells. F' is an adjacent section of C' stained with sense probe as control, which resulted in little signal. s: stromal; c: cancer. Scale bar= 100 um.

A set of 32 cancer-enriched and recurrent chimeric RNAs we recently discovered was chosen for initial screening [6]. To evaluate the expression of chimeric RNA, we performed RT-PCR using primer pairs tailored for each of the 32 chimeras so that each primer of the pair anneals to one parental gene therefore specifically amplifies the chimeric transcript but not the parental gene transcript. The assay was performed on several ESCC cell lines, including EC109, EC9706, HK2, HK3, and TE1, and compared to that of the immortalized normal esophageal epithelial cells such as NE1 and NE3 cells. These chimeric transcripts were confirmed by direct Sanger sequencing of RT-PCR products. In addition, we evaluated eighteen pairs of cancer/matched benign tissue isolated from ESCC patients. RT-PCR showed that out of the 32 chimeric RNAs, 18 displayed varying degrees of expression in cancer vs. matched benign tissue (Figure 2). However, we found conspicuously higher expression of 2 chimeras, namely *ASTN2-PAPPA* and *GOLM1-MAK10*, in patients' cancer tissue vs. matched benign tissue (Figure 2, 3A), as well as in ESCC cancer cell lines vs. immortalized esophageal cells (Figure 2, 3B). Among these two chimeras, we focused on *GOLM1-MAK10* in the subsequent studies for the following reasons: 1) the 5' parental gene *GOLM1* (also known as GOLPH2 or GP73), a Golgi membrane protein, is predominantly expressed in epithelial cells [14], the cell type that ESCC is derived from; and 2) the chimera *GOLM1-MAK10* retains most of the *GOLM1* sequence including the signal peptide/transmembrane domain that are required for secretion (see below and Figure 5). Active secretion is particularly important for cancer detection, considering that the most clinically useful cancer biomarkers to date, such as PSA for prostate cancer and CA125 for ovarian cancer, are secreted proteins.

**Figure 4:**
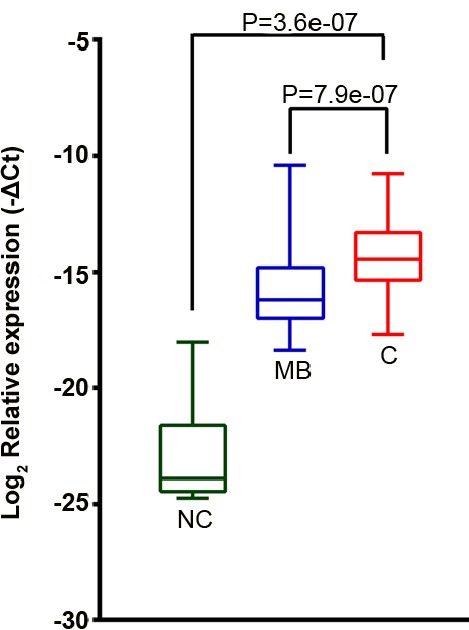
*GOLM1-MAK10* was expressed at a significantly higher level in ESCC The relative expression level of *GOLM1-MAK10* chimera as determined by quantitative RT-PCR (normalized to GAPDH). The plot compares the expression in esophageal epithelium of cancer (C, n=78), matched benign (MB, n=78), and an independent cohort of non-neoplastic esophageal epithelium from normal subjects (NC, n=10). *GOLM1-MAK10* was expressed at a significantly higher level in cancer vs. matched benign, or vs. non-neoplastic esophageal epithelium. p value of C vs. MB was calculated using paired student T test, while C vs. NC using unpaired student T test. Y-axis is (−ΔCt). Relative fold change in gene expression equals 2 to the power of (−ΔCt). Box-plot shows the range of relative expression levels with sample minimum and maximum represented by a short horizontal bar. The box itself represents 50% of all samples with the lower and upper quartile separated by a line that represents the median.

**Figure 5:**
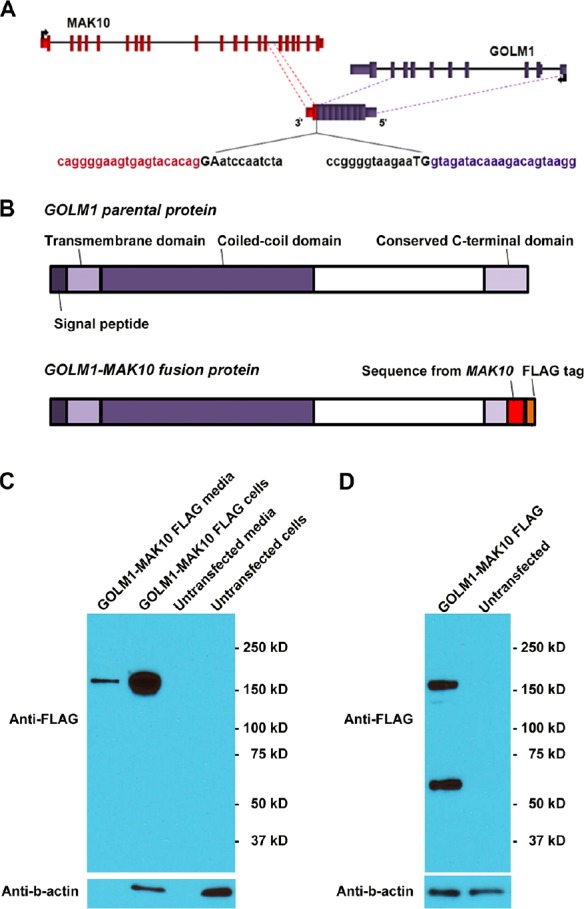
*GOLM1-MAK10* leads to the translation of a secreted fusion protein (A) Schematic of *GOLM1-MAK10* chimera. The chimera of *GOLM1-MAK10* could result in the substitution of the last 25 amino acids of *GOLM1* by 26 new amino acids with the 3' UTR from *MAK10*. In the pictogram, coding exons are represented by blocks connected by horizontal lines representing introns. The 5′- and 3′-UTR are represented by shorter blocks. Arrows indicate direction of transcription of parental genes. Dashed lines indicate the boundaries of the sequence contributing to chimeric RNAs. Splice junctions are shown as uppercase letters. The sequences in black are part of the introns removed in the formation of the chimeric RNAs. Note that a discernible 5' splice site and a 3' splice site are present at the RNA junction. (B) Chimera RNA *GOLM1-MAK10* is predicted to translate into a fusion protein with the majority of amino acid sequence of *GOLM1* retained, and has a near identical size to the *GOLM1* parental protein. *GOLM1-MAK10* would retain the signal peptide region and the transmembrane domain that are required for secretion. The coiled-coil domain required for the dimerization of *GOLM1* also remains unchanged. (C) *GOLM1-MAK10* construct with FLAG tag was expressed under the control of the CMV promoter in HEK 293T cells. Fusion protein is efficiently translated and secreted in the media as dimers. For FLAG western, 0.4% of the total cell extract and 0.4% of the total media were loaded. For Actin western, equal amounts of extracts were loaded. (D) Iodoacetamide treatment, which reduces the disulfide bonds, partially converted dimers into monomers. The expected size of the monomer is 44 kD. However, we observe bands at roughly 60 kD for the monomer, suggesting that they are heavily glycosylated. This is consistent with previous observations of *GOLM1*.

To determine the cellular origin of *GOLM1-MAK10* within patients' tumor, we performed *in situ* hybridization using antisense probes against the chimeric junctions and segments specific to *GOLM1-MAK10*. The results revealed strong expression in cancerous esophageal epithelium from ESCC patients with very low staining in the adjacent stromal cells (Figure 3C). This confirmed that the chimera in question is derived primarily from cancer cells within the tumor. The data hence suggest the association of the chimeric *GOLM1-MAK10* with ESCC.

### Validation of GOLM1-MAK10 in a large cohort of ESCC vs. matched benign tissues and non-neoplastic tissues

To validate the prevalence of *GOLM1-MAK10* in ESCC and determine its clinicopathological significance, a large cohort of paired cancer/matched benign tissues from a total of 115 patients were examined by quantitative RT-PCR analysis. As shown in Figure 4, the chimera *GOLM1-MAK10* was expressed at a distinctly higher level in cancer tissues vs. matched benign tissues (p=7.9e-07). Abberant *GOLM1-MAK10* was associated with histologic differentiation and lymph node metastasis (p=0.013 & 0.023 respectively, Table 1), but not statistically signifcant with any other clinicopathological indicators.

**Table 1:** Correlation between *GOLM1-MAK10* level and clinicopathologic variables in patients with ESCC

Variables	No. of patients	GOLM1-MAK10		
		Low (%)	High (%)	P*
Total samples	78	39 (50.0)	39 (50.0)	
Sex				
Male	60	31 (51.7)	29 (48.3)	0.591
Female	18	8 (44.4)	10 (55.6)	
Age (years)				
≤ 60	39	20 (51.3)	19 (48.7)	0.821
> 60	39	19 (48.7)	20 (51.3)	
Tumor location				
Upper/Middle	68	35 (51.5)	33 (48.5)	0.498
Lower	10	4 (40.0)	6 (60.0)	
Tumor size				
< 5 cm	18	8 (44.4)	10 (55.6)	0.591
≥ 5 cm	60	31 (51.7)	29 (48.3)	
Histologic differentiation				
Well	25	18 (72.0)	7 (28.0)	0.013
Moderate	35	16 (45.7)	19 (54.3)	
Poor	18	5 (27.8)	13 (72.2)	
Tumor depth				
T1/T2	14	5 (35.7)	9 (64.3)	0.238
T3/T4	64	34 (53.1)	30 (46.9)	
Lymph node metastasis				
N0	38	24 (63.2)	14 (36.8)	0.023
N1	40	15 (37.5)	25 (62.5)	
Stage				
I/II	42	21 (50.0)	21 (50.0)	1.000
III	36	18 (50.0)	18 (50.0)	

* As determined by chi-square test

To minimize the possible issues of tissue impurity caused by matched benign tissues that may contain multi-focal premalignant lesions that precede histological changes [15], 10 esophageal specimens obtained from subjects without esophageal neoplasia were examined. This refined analysis compares cancer tissue from ESCC patients directly to non-neoplastic tissue from normal subjects, thus issues of tissue impurity and premalignant lesions are minimized. The analysis revealed that non-neoplastic tissue from subjects without esophageal neoplasia expressed near undetectable level of chimera, as compared to the remarkably high level in ESCC tissue (p=3.7e-07, Figure 4). This marked difference in expression level suggests that *GOLM1-MAK10* could be a promising indicator to differentiate between ESCC patients and subjects without cancer.

### GOLM1-MAK10 encodes and leads to the translation of a secreted fusion protein

We next determined if *GOLM1-MAK10* translates into a secreted fusion protein. The RNA junction sequences determined by RT-PCR and Sanger sequencing enabled us to analyze the potential protein consequences of *GOLM1-MAK10*. Our analysis indicated that *GOLM1-MAK10* could result in the substitution of the last 25 amino acids of GOLM1 by 26 new amino acids and a new 3' UTR from *MAK10* (Figure 5A). This would leave the signal peptide and transmembrane domain of *GOLM1* intact [16], hence leading to the secretion of an aberrant fusion protein. To investigate this possibility, we cloned the full length ORF of *GOLM1-MAK10* and tagged it with a FLAG sequence (Figure 5B), and transfected it in HEK 293T cells. Protein analysis confirmed that *GOLM1-MAK10* is indeed translated into a fusion protein. The size of the fusion protein suggests a dimer formation in a manner similar to that of the parental *GOLM1* (Figure 5C and 5D). This is expected, because the coiled-coil domain required for the dimerization of *GOLM1* also remains unchanged in *GOLM1-MAK10*. Importantly, this fusion protein was also found in the cultured media devoid of cellular debris, indicating that it is secreted in a manner similar to its parental protein *GOLM1* (Figure 5C). Thus, the secreted fusion protein makes *GOLM1-MAK10* an attractive candidate as useful biomarker for detecting ESCC.

### Mechanisms responsible for generation of GOLM1-MAK10

**Figure 6:**
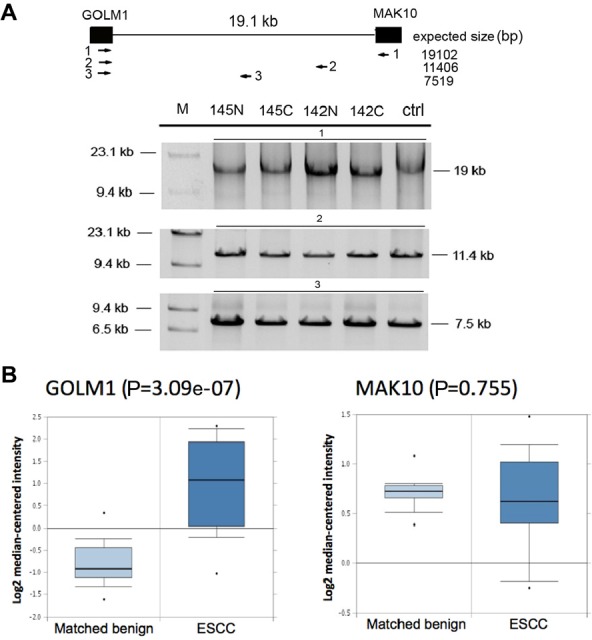
Chimeric *GOLM1-MAK10* is generated through transcription-induced mechanisms (A) Genomic DNA analysis of *GOLM1-MAK10* revealed no chromosomal rearrangement. This assay was possible for *GOLM1-MAK10* as parental genes are adjacent genes on the same chromosome. Upper panel: diagram of the intergenic region between the RNA junction of *GOLM1-MAK10*. Black boxes represent the chimeric RNA exons. Three primer pairs were used to scan the 19kb intergenic region. The expected product sizes are indicated below the diagram. Lower panel: results of long-range PCR. Results from two ESCC patients (145C and 142C), the matched benign esophageal tissues (145N and142N), and normal human genomic DNA from Promega (ctrl) were shown. Absence of size differences between cancer, benign, and normal control indicates that there were no gross DNA rearrangements. (B) The 5' parental gene *GOLM1* is over-expressed in ESCC. The microarray expression analysis was based on the published data by Hu et al 2010 available from Oncomine (17). The plot compares the expression in esophageal epithelium of matched benign (n=17) vs. esophageal epithelium of cancer (n=17). The 5' parental gene was over-expressed in cancer vs. matched benign (p value= 3.09e-07), but not the 3' parental gene (p value= 0.775). Y-axis represents relative gene expression in log2 scale. Box-plot shows the range of relative expression levels with sample minimum and maximum represented by a short horizontal bar. The box itself represents 50% of all samples with the lower and upper quartile separated by a line that represents the median. Dots outside the bar are considered outliers.

To determine if the chimera is the result of genomic DNA rearrangement (fusion gene), we performed long-range PCR on the genomic DNA isolated from patients' tissue that expressed the corresponding chimeric RNA. The assay yielded indistinguishable product sizes between ESCC tissue, matched benign tissue, and normal human genomic DNA control, indicating that there were no apparent DNA rearrangements in ESCC tissues (Figure 6A). Intriguingly, further sequence analyses indicated that *GOLM1-MAK10* RNA contains a discernable 5' splice site and a 3' splice site at the RNA junction (Figure 5A), indicating that the formation of these chimeras is mediated by RNA splicing. Collectively, the observed splice site at RNA junctions, and the lack of evidence of DNA-level rearrangements, together with the fact that these paired genes are adjacent genes in the genome, indicates that the chimera is most likely generated by read-through/splicing at RNA level.

Because the transcription of a chimeric RNA generated by read-through/splicing is controlled by the promoter of the 5' parental gene, the higher level of chimera in cancer raised the question if this is because that the 5' parental genes (*GOLM1*) is over-expressed in ESCC? To answer this question, we analyzed the available ESCC microarray gene expression database [17] in Oncomine. As shown in Figure 6B, the 5' parental gene *GOLM1* were over-expressed in cancer tissue vs. matched benign (fold change= 3.4) while the expression of 3' parental gene *MAK10* remained unchanged. Therefore, the elevated chimera level in ESCC cancer tissue could at least in part be the consequence of over-expression of 5' parental gene and increased read-through transcription in cancer.

## DISCUSSION

In this study, by employing multiple approaches we for the first time showed that *GOLM1-MAK10*, is highly enriched in ESCC. This chimera is likely generated through RNA processing and is unrelated to chromosomal rearrangements. Thus far, no recurrent fusion gene or chimeric RNAs have been identified in ESCC. Recurrent fusion genes have been described in many hematological cancers and solid tumors, and subsequently provided new insights into the molecular subtypes of cancers and pathogenesis. The biological consequences of chimeric RNAs however would be similar to that of gene fusions, as both mechanisms generate fusion transcripts that possess sequences from two parental genes. Transcription-induced chimeric RNAs thus, like fusion genes, could encode fusion proteins and contribute to oncogenesis. Our investigation demonstrated that chimeric RNAs are abundant in ESCC. In particular, *GOLM1-MAK10* was recurrent among patients, and was enriched in tumor tissues at readily detectable levels, while near undetectable in normal esophageal tissue from subjects without esophageal neoplasia. This points to the important potential utility of *GOLM1-MAK10* as molecular signatures for identifying ESCC and for early detection. In prostate cancer, the chimeric RNA *SLC45A3-ELK4* is present in urine at detectable levels [7, 8]. This suggests that *GOLM1-MAK10* could also be released by ESCC tumor into esophageal mucus or saliva protected by exosomes, thus serve as a non-invasive biomarker detectable using simple PCR-based assay.

Interestingly, our cell culture results demonstrated that chimera *GOLM1-MAK10* identified in ESCC led to the translation of potentially functional fusion protein in human cells. *GOLM1* (also known as GOLPH2 or GP73) is a Golgi membrane protein. The expression of *GOLM1* gene is predominantly in epithelial cells [14], the cell type that ESCC is derived from. Previous reports indicated that elevated expression of parental *GOLM1* is associated with localized prostate cancer [18] and HER-2/neu proto-oncogene overexpression [19]. In addition, *GOLM1* protein was found at high levels in the serum of patients with hepatocellular carcinoma [20, 21]. While *GOLM1* was reported as a promising diagnostic cancer marker, its biological function is unclear [20, 21]. Our observation revealed a close correlation between aberrant *GOLM1-MAK10* and lymph node metastasis as well as tumor differentiation, implying a potential function in ESCC. The chimera *GOLM1-MAK10* retains most of the *GOLM1* sequence including the signal peptide region and the transmembrane domain that are required for targeting *GOLM1* to the Golgi complex for secretion. This feature of secretion is particular important, considering that the most clinically useful cancer biomarkers to date, such as PSA for prostate cancer and CA125 for ovarian cancer, belong to the class of secreted proteins. Our findings that the aberrant chimera *GOLM1-MAK10* was enriched in ESCC, and that *GOLM1-MAK10* produced a secreted fusion protein, suggests the possibility of a unique protein signature detectable by standard non-invasive ELISA assays.

Intriguingly, approximately 18 of the 32 recurrent chimeric RNAs identified in prostate cancer were also present in ESCC (Figure 2), suggesting that similarity exists between the two cancer types. However, in ESCC the chimera *GOLM1-MAK10* appears to be highly enriched while the two most prominent prostate cancer chimeric RNA, *SLC45A3-ELK4* and *TMEM79-SMG5* [6], and the fusion transcript from the prostate cancer fusion gene *TMPRSS2-ERG* [22], are undetectable in ESCC (Figure 2), indicating that the different cancer types posses different prominent molecular signatures with respect to chimeric RNA species.

*GOLM1* is overexpressed in androgen-responsive Leydig cell tumor and prostate cancer [18, 23]. We performed bioinformatics analysis (http://alggen.lsi.upc.es/cgi-bin/promo_v3/promo/promoinit) and found that *GOLM1* promoter region [14] spanned six androgen-responsive elements (AREs). It is known that hormones modulate fusion genes and their RNA products. For example, the expression of both chimeric *TMPRSS2-ERG* and *SLC45A3-ELK4* are androgen-regulated in prostate cancer [7, 8], a male and androgen-sensitive cancer. Given that ESCC occurs predominantly in males and that sex steroid pathways such as androgen signaling may be involved in ESCC [24], we hypothesized that the expression of chimeric *GOLM1-MAK10* may also be modulated by androgen. Our preliminary data suggest that androgen treatment of ESCC cell lines quickly elevated the expression of endogenous *GOLM1-MAK10* within 12 hours, and maintained up to 48 hours, while the expression of *GOLM1-MAK10* in untreated cells were unchanged during the same experimental period, indicating the regulation of the chimera expression is participated by androgen signaling (Supplementary Figure 1). The possibly hormone-dependent regulation of chimeric RNAs, once further elucidated, may better our understanding of molecular mechanisms responsible for ESCC carcinogenesis and potentially provides novel target pathways for therapeutic intervention.

In summary, this investigation shows that chimeric RNA, previously unknown in esophageal carcinoma, is prevalent in ESCC, and identified *GOLM1-MAK10* as important molecular signature of ESCC. Chimeric RNAs may represent a novel class of molecular alteration in ESCC that provides important insights into the molecular mechanism invovled in ESCC. Continued research of chimeric RNAs, in particular *GOLM1-MAK10*, will augment this and potentially provide novel target pathways for future therapies. The aberrant secreted fusion protein translated from GOLM1-MAK10 could serve as a unique protein signature detectable by standard non-invasive assays. This is critical, given that no clinically useful biomarker is currently available for screening this deadly disease or monitoring the treatment response.

## MATERIAL AND METHODS

### Cell lines and human ESCC specimens

The cell lines EC109, EC9706 (obtained from the Cell Bank of the Chinese Academy of Sciences, China), TE1 (kindly provided by Dr. X.C. Xu at UT M.D. Anderson Cancer Center, USA), and HKESC-2, and HKESC-2, HKESC-3, NE1 and NE3 cells (kindly provided by Dr. S.W. Tsao, University of Hong Kong) were prepared as described [11, 12]. All human samples were obtained from cancer hospital of Shantou University Medical College in the Chaoshan littoral region located in southern China, which is recognized as one of the regions with the highest incidence of esophageal carcinomas in China. Study was approved by the institution ethics committee and specimens were collected with informed consent under an Institutional Review Board–approved protocol (IRB protocol number: #04-070). A summary of clinicopathological information of patients is provided in Supplementary Table 1.

### RT-PCR, direct DNA sequencing and qRT-PCR

Total RNA was extracted from tissues and cell lines using TRIzol reagent as per manufacturer's protocol (Invitrogen). RT-PCR was performed in human samples and cells as described previously [6]. The products of RT-PCR were gel purified and sequenced by Sanger sequencing to identify the exact fusion junctions of the candidate events [6]. qRT-PCR was performed using Absolute Blue QPCR SYBR Green low ROX mix (Thermo Scientific) on Applied Biosystems' 7500 real-time PCR system as described previously [6]. All assays were performed in triplicate and the results are shown as average fold change relative to GAPDH, which serves as an internal control. Primers used for qRT-PCR are listed in the online Supporting Information.

### In situ hybridization

Nonradioactive in situ hybridization was performed as described previously [13]. Briefly, antisense RNA probes labeled with digoxigenin were transcribed from cDNA templates. Paraffin-fixed tissue sections from ESCC were deparaffinized, treated with proteinase K, pre-hybridized and then hybridized with probes. Following hybridization, the slides were washed and treated with a blocking reagent and then incubated with anti-digoxigenin and detected as detailed [13]. Antisense probes were designed against the fusion junction or RNA segments specific to these chimeric RNAs, so as to distinguish chimeric RNAs from the parental mRNAs. We tested and confirmed the specificity of the antisense probes to distinguish chimeric RNAs from parental mRNA using dot blot assay. Antisense probes used for *in situ* hybridization are listed in online Supporting Information.

### Long-range PCR

LA PCR kit (Takara) was used for long-range PCR analysis of patient genomic DNA as described [6]. To ensure that the PCR primers designed can amplify the intended DNA fragment, we used commercial human genomic DNA (Promega) as a control. Primers used are listed in the online Supporting Information.

### Cloning and transfection of chimera

Using the primers (listed in the online Supporting Information), RT-PCR was performed on patient samples to generate the full length *GOLM1-MAK10*. A FLAG tag was then added to the C-terminus of this PCR product and cloned into the vector HDM. HEK-293T cells were transfected with this plasmid using TransIT-293 reagent (Mirus) according to the manufacturer's protocol.

### Western blotting

48 hours after transfection of cloned full-length *GOLM1-MAK10*, proteins within transfected cells were extracted using RIPA buffer (Santa Cruz Biotechnology). Western blotting was performed using the following antibodies: Anti-FLAG (SIGMA Catalog number F1804) and Anti-β-actin (SIGMA Catalog number A2228).

## Supplementary Methods, Figures and Tables






