# Supplementary material for: Aberrant chimeric RNA GOLM1-MAK10 encoding a secreted fusion protein as a molecular signature for human esophageal squamous cell carcinoma
Source: Oncotarget. 2013 Nov 1;4(11):2135–43. doi: 10.18632/oncotarget.1465 (PMC3875775; doi:10.18632/oncotarget.1465)
Supplement: Supplementary file 1 [file oncotarget-04-2135-s001.pdf]

Aberrant chimeric RNA *GOLM1-MAK10* encoding a secreted fusion protein as a molecular signature for human esophageal squamous cell carcinoma - Zhang et al

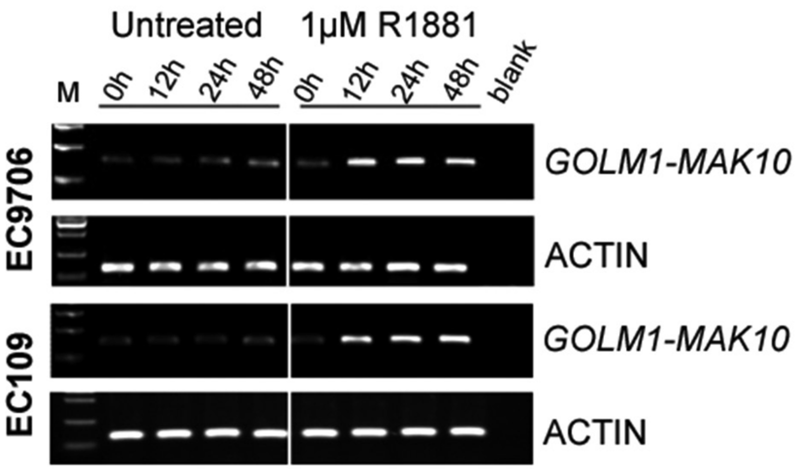

Supplementary Figure 1: Androgen treatment upregulates the expression of *GOLM1-MAK10* RNA. Two ESCC cell lines, EC9706 and EC109, were treated with R1881 for various durations followed by RT-PCR assay. β-actin was used as the internal control. Upregulated as early as 12 h following R1881 treatment.
