# Supplementary material for: Aberrant chimeric RNA GOLM1-MAK10 encoding a secreted fusion protein as a molecular signature for human esophageal squamous cell carcinoma
Source: Oncotarget. 2013 Nov 1;4(11):2135–43. doi: 10.18632/oncotarget.1465 (PMC3875775; doi:10.18632/oncotarget.1465)
Supplement: Supplementary file 2 [file oncotarget-04-2135-s002.doc]

| **Patient ID** | **Race** | **Age** | **Sex** | **TNM Stage** |
| --- | --- | --- | --- | --- |
| 66 | Ethnic Chinese | 63 | Male | T3 N0 M0, IIA |
| 67 | Ethnic Chinese | 54 | Male | T2 N1 M0, IIIA |
| 79 | Ethnic Chinese | 57 | Male | T3 N0 M0, IIA |
| 82 | Ethnic Chinese | 75 | Male | T4 N1 M0, IIIB |
| 83 | Ethnic Chinese | 57 | Male | T2 N0 M0, IIA |
| 87 | Ethnic Chinese | 65 | Male | T4 N1 M0, IIIA |
| 107 | Ethnic Chinese | 75 | Female | T3 N1 M0, IIIA |
| 109 | Ethnic Chinese | 58 | Male | T3 N1 M0, IIIB |
| 111 | Ethnic Chinese | 72 | Female | T3 N0 M0, IIA |
| 114 | Ethnic Chinese | 56 | Male | T4 N0 M0, IIA |
| 131 | Ethnic Chinese | 60 | Male | T4 N1 M0, IIIC |
| 143 | Ethnic Chinese | 54 | Male | T4 N1 M0, IIIC |
| 145 | Ethnic Chinese | 73 | Male | T2 N1 M0, IIB |
| 170 | Ethnic Chinese | 64 | Female | T3 N0 M0, IIA |
| 172 | Ethnic Chinese | 57 | Male | T3 N1 M0, IIIA |
| 173 | Ethnic Chinese | 58 | Male | T3 N0 M0, IIB |
| 176 | Ethnic Chinese | 70 | Male | T3 N1 M0, IIB |
| 182 | Ethnic Chinese | 61 | Male | T2 N0 M0, IIB |
| 183 | Ethnic Chinese | 61 | Male | T2 N1 M0, IIA |
| 184 | Ethnic Chinese | 57 | Male | T3 N0 M0, IIB |
| 188 | Ethnic Chinese | 62 | Male | T4 N0 M0, IIB |
| 190 | Ethnic Chinese | 38 | Male | T3 N1 M0, IIIA |
| 193 | Ethnic Chinese | 62 | Male | T3 N3 M0, IIIC |
| 195 | Ethnic Chinese | 46 | Female | T4 N0 M0, IIB |
| 196 | Ethnic Chinese | 63 | Female | T2 N0 M0, IIB |
| 197 | Ethnic Chinese | 40 | Male | T3 N2 M0, IIIB |
| 199 | Ethnic Chinese | 71 | Female | T3 N0 M0, IIA |
| 200 | Ethnic Chinese | 70 | Female | T3 N0 M0, IIA |
| 201 | Ethnic Chinese | 70 | Male | T3 N0 M0, IIA |
| 202 | Ethnic Chinese | 71 | Male | T3 N0 M0, IIA |
| 203 | Ethnic Chinese | 62 | Male | T4 N0 M0, IIB |
| 204 | Ethnic Chinese | 38 | Female | T2 N0 M0, IB |
| 205 | Ethnic Chinese | 55 | Male | T2 N1 M0, IIB |
| 207 | Ethnic Chinese | 69 | Male | T4 N2 M0, IIIB |
| 210 | Ethnic Chinese | 54 | Male | T3 N2 M0, IIIB |
| 213 | Ethnic Chinese | 52 | Male | T3 N0 M0, IIA |
| 214 | Ethnic Chinese | 50 | Female | T2 N0 M0, IB |
| 215 | Ethnic Chinese | 76 | Male | T3 N0 M0, IIA |
| 216 | Ethnic Chinese | 78 | Female | T4 N0 M0, IIA |
| 217 | Ethnic Chinese | 60 | Male | T2 N0 M0, IB |
| 218 | Ethnic Chinese | 47 | Male | T4 N1 M0, IIIA |
| 219 | Ethnic Chinese | 75 | Male | T3 N0 M0, IIB |
| 222 | Ethnic Chinese | 44 | Male | T4 N3 M0, IIIC |
| 223 | Ethnic Chinese | 56 | Male | T4 N0 M0, IIB |
| 224 | Ethnic Chinese | 60 | Female | T4 N0 M0, IIB |
| 225 | Ethnic Chinese | 59 | Male | T4 N0 M0, IIIC |
| 226 | Ethnic Chinese | 62 | Female | T3 N1 M0, IIB |
| 227 | Ethnic Chinese | 48 | Male | T3 N0 M0, IIA |
| 232 | Ethnic Chinese | 60 | Male | T3 N0 M0, IIB |
| 233 | Ethnic Chinese | 51 | Male | T4 N1 M0, IIB |
| 234 | Ethnic Chinese | 59 | Male | T3 N0 M0, IIB |
| 235 | Ethnic Chinese | 71 | Female | T3 N0 M0, IIIB |
| 237 | Ethnic Chinese | 62 | Male | T4 N1 M0, IIIA |
| 238 | Ethnic Chinese | 66 | Female | T3 N0 M0, IIA |
| 239 | Ethnic Chinese | 69 | Male | T1 N0 M0, IIA |
| 240 | Ethnic Chinese | 72 | Female | T3 N1 M0, IIIA |
| 241 | Ethnic Chinese | 64 | Male | T4 N3 M0, IIIC |
| 242 | Ethnic Chinese | 68 | Female | T4 N1 M0, IIIA |
| 244 | Ethnic Chinese | 53 | Male | T3 N0 M0, IIB |
| 245 | Ethnic Chinese | 64 | Male | T3 N0 M0, IIA |
| 246 | Ethnic Chinese | 61 | Male | T4 N3 M0, IIIC |
| 248 | Ethnic Chinese | 65 | Female | T3 N0 M0, IIB |
| 249 | Ethnic Chinese | 64 | Male | T4 N0 M0, IIA |
| 315 | Ethnic Chinese | 75 | Male | T4 N0 M0, IIA |
| 316 | Ethnic Chinese | 45 | Male | T3 N0 M0, IIB |
| 317 | Ethnic Chinese | 64 | Male | T3 N1 M0, IIIA |
| 318 | Ethnic Chinese | 60 | Male | T3 N0 M0, IIB |
| 319 | Ethnic Chinese | 58 | Male | T4 N3 M0, IIIC |
| 320 | Ethnic Chinese | 53 | Male | T3 N3 M0, IIIB |
| 321 | Ethnic Chinese | 53 | Male | T3 N0 M0, IIA |
| 322 | Ethnic Chinese | 48 | Male | Unknown |
| 326 | Ethnic Chinese | 57 | Male | T4 N1 M0, IIIA |
| 327 | Ethnic Chinese | 60 | Male | T3 N3 M0, IIIC |
| 328 | Ethnic Chinese | 60 | Male | T1 N0 M0, IB |
| 329 | Ethnic Chinese | 57 | Female | T1 N0 M0, IB |
| 330 | Ethnic Chinese | 69 | Male | T3 N0 M0, IIB |
| 331 | Ethnic Chinese | 54 | Male | T3 N0 M0, IIB |
| 333 | Ethnic Chinese | 66 | Male | T2 N0 M0, IIA |
| 334 | Ethnic Chinese | 46 | Male | T2 N0 M0, IB |
| 335 | Ethnic Chinese | 54 | Male | T3 N2 M0, IIIC |
| 336 | Ethnic Chinese | 45 | Male | T2 N1 M0, IIB |
| 337 | Ethnic Chinese | 63 | Male | T4 N0 M0, IIB |
| 338 | Ethnic Chinese | 42 | Male | T4 N3 M0, IIIC |
| 339 | Ethnic Chinese | 65 | Female | T3 N3 M0, IIIC |
| 340 | Ethnic Chinese | 59 | Male | T3 N3 M0, IIIC |
| 341 | Ethnic Chinese | 66 | Male | T3 N0 M0, IIB |
| 342 | Ethnic Chinese | 60 | Male | T4 N0 M0, IIB |
| 343 | Ethnic Chinese | 57 | Male | T3 N2 M0, IIIC |
| 344 | Ethnic Chinese | 47 | Male | T3 N2 M0, IIIB |
| 346 | Ethnic Chinese | 64 | Female | T4 N0 M0, IIB |
| 347 | Ethnic Chinese | 46 | Male | T3 N1 M0, IIIA |
| 348 | Ethnic Chinese | 44 | Male | T2 N2 M0, IIIA |
| 349 | Ethnic Chinese | 72 | Female | T3 N1 M0, IIIA |
| 383 | Ethnic Chinese | 66 | Male | T3 N3 M0, IIIC |
| 384 | Ethnic Chinese | 52 | Male | T3 N2 M0, IIIB |
| 386 | Ethnic Chinese | 61 | Male | T4 N2 M0, IIIB |
| 387 | Ethnic Chinese | 47 | Male | T3 N2 M0, IIIB |
| 389 | Ethnic Chinese | 65 | Male | T3 N0 M0, IIB |
| 390 | Ethnic Chinese | 55 | Male | T4 N3 M0, IIIC |
| 391 | Ethnic Chinese | 54 | Male | T3 N0 M0, IIA |
