# Supplementary material for: Aberrant chimeric RNA GOLM1-MAK10 encoding a secreted fusion protein as a molecular signature for human esophageal squamous cell carcinoma
Source: Oncotarget. 2013 Nov 1;4(11):2135–43. doi: 10.18632/oncotarget.1465 (PMC3875775; doi:10.18632/oncotarget.1465)
Supplement: Supplementary file 3 [file oncotarget-04-2135-s003.doc]

**Supplementary materials and methods**

**1. Primers and probes used**

**1). *Primers used for quantitative real-time PCR***

| Primers | Sequences | Product Size |
| --- | --- | --- |
| *GOLM1-MAK10*-forward | 5'-CAGGGAGAAACCAGCAGAAACTGA-3' | 154bp |
| *GOLM1-MAK10*-reverse | 5’-AAGGCTGCATCTTAGTTGTCCC-3’ |  |
| *GAPDH*-forward | 5’-TGCACCACCAACTGCTTAGC-3’ | 87bp |
| *GAPDH*-reverse | 5’-GGCATGGACTGTGGTCATGAG-3’ |  |
| ß-actin-forward | 5’-GAACCCCAAGGCCAACCGCGAGA-3’ | 149bp |
| ß-actin-reverse | 5’-TGACCCCGTCACCGGAGTCCATC-3’ |  |

***2).*** Primers used for Long-range PCR

|  | No. | Forward | Reverse |
| --- | --- | --- | --- |
| *GOLM1-MAK10* | 1 | GTGCTAATTTTACAGGGAGAAAC | TAGTTGTCCCCTTCACTCATG |
|  | 2 | GTGCTAATTTTACAGGGAGAAAC | GACCCAACTCTTAGAGGGCA |
|  | 3 | GTGCTAATTTTACAGGGAGAAAC | CACAGCCCTCTCCATTGTTG |

**3). Primers used for cloning chimera *GOLM1-MAK10:***

Eco-Sac-Not-Kozak-*GOLM1* forward:

5’ AAGAATTCCGCGGGCGGCCGCCATGATGGGCTTGGGAAAC 3’

G-M reverse:

5’CCCAAGCTTTTATCACTTGTCGTCATCGTCTTTGTAGTCGTTGTCCCCTTCACTCATGTGTCCATCTATGTTTCTGTCATTCCCTGCCAGGGCTGCTTGC 3’

**4). Antisense probe specific to *GOLM1-MAK10* but not to parental gene *GOLM1* or *MAK10* used for in situ hybridization:**

(gtaatacgactcactatagg)TCAAACCGTGGTGCTGAGGATTCACTTCAGGAGCTTTGGAGTTGATAAAAGATGATCTTCGGCAGAGTAGTTCTTCCCTTTCACTTGCGGACAAAATAAACCTGATATTTGAGAAGAAAAGGCTGCATCTTAGTTGTCCCCTTCACTCATGTGTC*CATCTATG

**2. Statistical Analysis for patient samples**

Log transformed expression values were median centered over each sample. For gene the normalization, ranking of expression values and median splits were done separately in each sample. Associations of GOLM1-MAK10 mRNA expression with histoclinical features were assessed with chi-square test.

**3. Androgen treatment**

For induction of chimera RNA by androgen, cells were cultured in 10% charcoal-stripped serum (Hyclone) containing phenol-red free media for 48 hours. Cells were treated with the synthetic androgen methyltrienolone (R1881) (Perkin-Elm) dissolved in absolute ethanol at a concentration of 1uM or without R1881 for 12, 24 and 48 respectively. Cells were then harvested for RT-PCR assays.
